# Finger avulsion injuries: A report of four cases

**DOI:** 10.4103/0019-5413.40259

**Published:** 2008

**Authors:** N Fejjal, R Belmir, S El Mazouz, NE Gharib, A Abbassi, AM Belmahi

**Affiliations:** Plastic Surgery-Hand Surgery Unit, IBN SINA Hospital, Rabat, Morocco

**Keywords:** Amputation, avulsion, digit, ray, replantation, ring

## Abstract

Injury that occurs to a finger wearing a ring though rare can have grave consequences. It is a preventable injury which has a peculiar mode of trauma that is usually occupational. Injury ranges from simple contusion to degloving of soft tissues to traumatic amputation. We hereby report our experience of four cases of finger avulsion injuries due to a ring and discuss their variable clinical presentation and individualized management.

## INTRODUCTION

Finger avulsion is a rare and grave injury. Injury caused to the finger wearing a ring by avulsion of the soft tissues, when the ring is pulled forcefully can cause a wide spectrum of damage ranging from a simple contusion injury to a traumatic amputation. Despite the high rate of failure,^1^,^2^ the literature contains enough evidence to support attempts to reconstruct these injuries.^3^,^4^ The authors report their experience in this injury through four cases and discuss its management.

## CASE REPORTS

### Case 1

**Figure 1:**
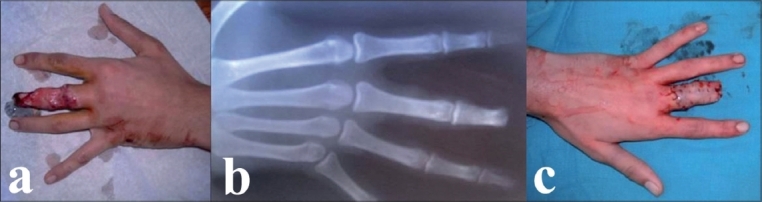
(a) Urbaniak Class III avulsion at the level of the DIP joint of left middle ray proximal phalanx. (b) Anteroposterior (AP) X-ray of left hand showing the level of amputation. (c) Postoperative appearance after skin graft

A 35-year-old male, refuse collector, presented with avulsion amputation of the left third digit at the level of the distal interphalangeal joint [Figure 1]. While rising from the sitting position inside the truck, his wedding ring got entangled in an iron hook of a metal piece in the floor of the truck. The case was defined as third stage according to Urbaniak classification.^5^ The patient reached the hospital 5 days after the accident. The stump was treated by full thickness skin grafting, after slight bone shortening.

### Case 2

**Figure 2:**
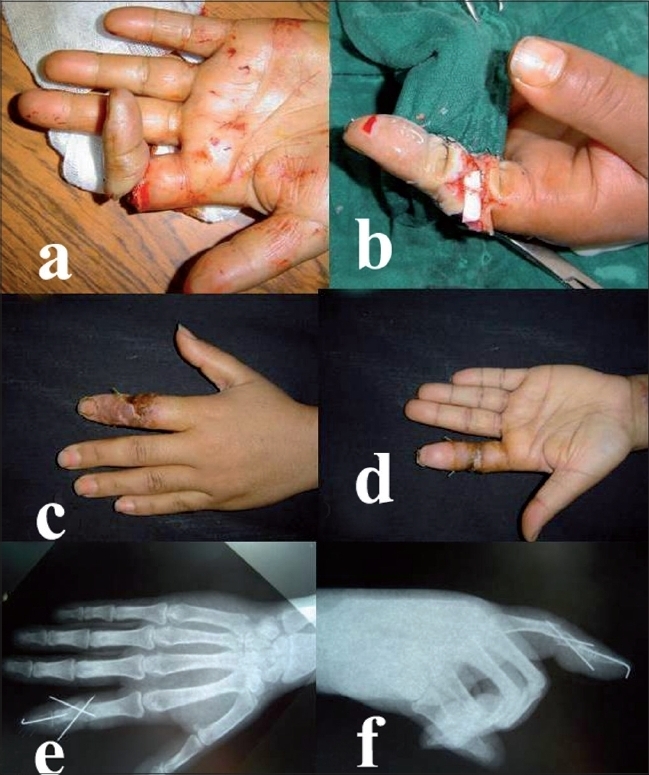
(a) Urbaniak Class III avulsion through proximal interphalangeal joint of left index finger with finger ischemia. Termino terminal anastomosis on the radial collateral artery after shortening arthrodesis of the proximal interphalangeal joint. Two weeks postoperative (c-d) aspect of the left hand after the procedure. Postoperative AP (e) and lateral (f) X-rays of left middle ray

A 31-year-old female, working as a waitress in a café, suffered an accident at her workplace. The patient's left index wedged into an orifice at the base of a refrigerator which she was trying to lift and move. This led to an avulsion of the second left digit and sub-amputation through the proximal interphalangeal joint (PIP) [Figure 2] with only an intervening cutaneous bridge remaining. The flexor digitorum profondus and superficial tendons were cut in Zone 2 and extensor tendon in Zone 4. There was no digital circulation. The case was defined as third stage according to Urbaniak classification. The patient was admitted to the hospital four hours after the accident. The patient was taken up for urgent wound irrigation and debridment. The cartilage of the head of the proximal phalanx of the index finger (P1) was destroyed. A shortening arthrodesis of the PIP joint was performed at 15° flexion. The flexor digitorum profondus tendon and extensor tendon were sutured end to end. Peroperative examination revealed contusion of the nerve and thrombosis of the digital artery on the ulnar aspect. Arterial repair was performed by anastomosis of the collateral radial artery. The venous flow was restored by placing an interposition vein graft between two dorsal veins, harvested from the palmar side of the subcutaneous tissue of the wrist. The collateral nerve at the radial aspect was repaired by simple suture. The skin could be closed primarily by simple interrupted sutures as the soft tissues were lax due to the shortening arthrodesis of the PIP joint. During the postoperative period there was a slight marginal skin necrosis of a part of the dorsal surface skin which healed secondarily by ointment dressings. On follow-up one year later, the patient was extremely satisfied with the reconstruction. She had an active range of motion of 90° at the metacarpophalangeal (MCP) joint and good return of protective sensation.

### Case 3

**Figure 3:**
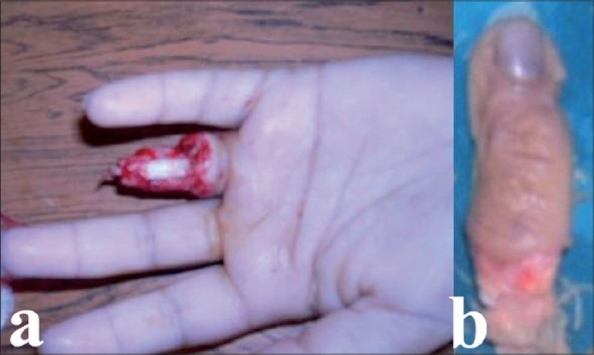
Urbaniak Class III avulsion at the level of the PIP joint of left fourth finger (a) also depicting the crushed amputated digit (b)

A 24-year-old right-handed male carpenter, presented with avulsion amputation of the right fourth digit at the level of P1 resulting from an occupational accident. While fabricating a wooden piece, his wedding ring was caught by the wood-turning lathe. This led to an amputation at the level of the proximal phalanx with a section of the extensor and flexor tendons. The amputated digit was crushed [Figure 3]. The case was defined as third stage according to Urbaniak classification. The patient was admitted to the hospital two hours after the accident where the stump was surgically treated. Considering the state of the amputated part and the fact that the patient was a manual worker, we decided against replantation.

### Case 4

**Figure 4:**
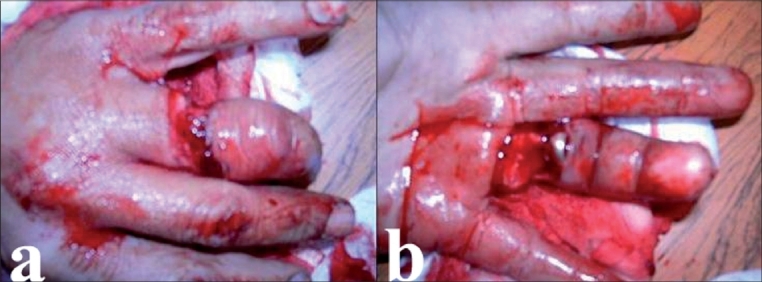
Urbaniak Class I avulsion showing cut extensor apparatus of the left fourth finger [dorsal (a) and volar (b) view].

A 45-year-old male farmer, presented with an occupational injury. While driving in a cow into a cowshed, his wedding ring accidentally got entangled into the rope tied around the cow. The resultant sudden violent forward thrust by the cow led to a circumferential lesion formation at the base of his left fourth digit, without disconnection [Figure 4]. The case was defined as first stage according to Urbaniak classification. The wedding ring was removed and the clinical examination revealed no signs of arterial or neurological damage. After careful irrigation and debridment, the extensor tendon was sutured and the wound was closed. Antibiotic and analgesic therapy was administered and the hand was kept elevated. Postoperatively, a slight edema was observed. It had not compromised the arterial and venous flow of the digit and disappeared within a period of several days. The patient regained good hand function with a slight loss of active flexion of the fourth left digit.

## DISCUSSION

Ring avulsion injuries often present a technical challenge. The goal is to salvage, maintain function and if possible, provide an esthetic appearance. They result from the mechanism of crushing, shearing and avulsion, inducing severe macroscopic and microscopic damage.^6^

Before the advent of microsurgery, many techniques were used to cover the denuded finger, such as use of tubed pedicle flaps from abdominal wall, but the results were often unsatisfactory.^7^ Many classification systems have been proposed to provide a more rational approach for the management of these injuries. 7–11 Among them, the classification of Urbaniak and colleagues is one of the best known as a scheme to grade the injury. It classifies lesions into three stages [Table 1].

**Table 1:** Urbaniak classification

Class I	Circulation adequate. Standard bone and soft tissue treatment is sufficient
Class II	Circulation inadequate. Vessel repair preserves viability permitting immediate or delayed repair of other tissues
Class III	Complete degloving or completed amputation. Judgement is essential because, although a complete amputation can be revascularised and viability restored, the potential for function is limited. In degloving injuries, the potential for useful function exists, but revascularisation is not easy or may not be possible

Although management of Class I cases is simple, Class II ring avulsions require some type of vascular repair because the circulation is inadequate in contrast to Class I cases. However, the most difficult treatment is related to a Class III ring avulsion in which there is complete degloving or amputation.

Several authors agree that replantation of completely amputated finger avulsions is often unsuccessful because of vascular damage involving a long segment of the artery.^12^,^13^ Inadequate debridement of the artery induces failures. Furthermore, it has been stated that when the arteries are disrupted upto the digital pulp, replantation is likely to lead to a failure.^14^

In Class III lesions with an intact PIP joint and flexor digitorum superficialis tendon reimplantation should be tried. However, we think that a ray amputation is a better alternative, if the PIP joint is damaged.^4^,^14^,^15^

In Patient 2 who presented with a third stage according to Urbaniak classification, reimplantation was done in spite of a sub-amputation through proximal phalanx because she refused amputation considering the esthetic aspect. She was explained well regarding the likely loss of function of the ray and the chances of developing postoperative gangrene with reimplantation.

In Case 1 reimplantation could have been a better option if the patient had sought medical attention early (< 24 h) with proper preservation of the amputated digit. He refused ray amputation, hence we debrided the stump and covered it with skin graft. This case shows that the Moroccan population needs to be sensitized more about hand injuries and that amputated rays and digits etc can be reimplanted provided the patient and the part reaches the caregiver timely and in an appropriate condition.

For digital artery repair we used vein graft. We insisted on the quality of debridement of the arterial ends for successful revascularisation.^14^ Some surgeons practice rerouting of an arterial pedicle from an adjacent digit but that technique results in the sacrifice of a major artery of the digit and a donor site problem as well.^15^ Our approach is to anastomose two or three veins end to end when possible or use an interpositional venous graft and close the skin defect using small local flaps or full-thickness skin grafts.^7^ However, we must follow the patient carefully for a long time as in any microsurgery procedure and be aware of the possibility of occurrence of a late arterial failure.^16^

## CONCLUSION

Ring finger avulsion injuries are very rare. We insist on prevention, especially in occupations involving manual and hand work. Ring must be removed from the finger before working. Microsurgery is superior to any method of primary or secondary reconstruction from a functional and esthetic point of view.
